# Genotype to Phenotype Mapping and the Fitness Landscape of the *E. coli lac* Promoter

**DOI:** 10.1371/journal.pone.0061570

**Published:** 2013-05-01

**Authors:** Jakub Otwinowski, Ilya Nemenman

**Affiliations:** 1 Department of Physics, Emory University, Atlanta, Georgia, United States of America; 2 Department of Biology, University of Pennsylvania, Philadelphia, Pennsylvania, United States of America; 3 Department of Physics, Department of Biology, and Computational and Life Sciences Initiative, Emory University, Atlanta, Georgia, United States of America; Albert Einstein College of Medicine, United States of America

## Abstract

Genotype-to-phenotype maps and the related fitness landscapes that include epistatic interactions are difficult to measure because of their high dimensional structure. Here we construct such a map using the recently collected corpora of high-throughput sequence data from the 75 base pairs long mutagenized *E. coli lac* promoter region, where each sequence is associated with its phenotype, the induced transcriptional activity measured by a fluorescent reporter. We find that the additive (non-epistatic) contributions of individual mutations account for about two-thirds of the explainable phenotype variance, while pairwise epistasis explains about 7% of the variance for the full mutagenized sequence and about 15% for the subsequence associated with protein binding sites. Surprisingly, there is no evidence for third order epistatic contributions, and our inferred fitness landscape is essentially single peaked, with a small amount of antagonistic epistasis. There is a significant selective pressure on the wild type, which we deduce to be multi-objective optimal for gene expression in environments with different nutrient sources. We identify transcription factor (CRP) and RNA polymerase binding sites in the promotor region and their interactions without difficult optimization steps. In particular, we observe evidence for previously unexplored genetic regulatory mechanisms, possibly kinetic in nature. We conclude with a cautionary note that inferred properties of fitness landscapes may be severely influenced by biases in the sequence data.

## Introduction

Many aspects of evolution, such as selection, recombination, and speciation, depend on the relationships between genotype, phenotype, and fitness. These relationships often involve complex and collective effects [1], which are difficult to untangle. One approach is to measure the fitness of many different genotypes, and build a *fitness landscape*, a high dimensional map from genotype/phenotype to reproductive fitness. This concept was first introduced by Sewell Wright in 1932 [2]. Evolutionary dynamics and adaptation depend crucially on features of the fitness landscape, and many studies have quantified large scale features of landscapes, including genetic interactions [3]–[10], the presence of stabilizing selection [11], [12], or the reproducibility of evolutionary paths [7], [13].

A major difficulty that has precluded mapping of large fitness landscape, is *epistasis*, which is the dependence of fitness effects of a mutation on the presence of other mutations. Epistasis makes the inference of landscapes combinatorially complex. This problem has attracted substantial attention. For example, millions of interactions between gene pairs have been measured from genetic knockout experiments [14]–[19]. Higher order epistatic interactions, that is those involving more than two loci at a time, have also been investigated for small fitness landscapes [3].

Another popular approach is mapping genotypes to phenotypes (also known as the Quantitative trait loci or QTL analysis [20]), which includes the dimensionality reduction problem, but is simpler since many phenotypes are easier to quantify reliably than the number of progenies, which exhibits large fluctuations. One then separately studies the lower dimensional map from the phenotype to the reproductive rate to complete the construction of the fitness landscape.

Unfortunately, few of these pioneering studies have provided a genotype to phenotype or to fitness mapping for longer genetic sequences, and most such large maps are modeled without epistasis (see, e. g., [21]). Indeed, a complete landscape would be defined not by genes or specific loci, but by all possible nucleotide sequences. However with 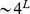 different sequences of length 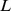, it had been impractical to measure the landscapes for sequences of relatively large length until next generation sequencing technologies dramatically lowered the cost [22]. Nonetheless, measuring phenotypes of a large number of sequences is still tricky, and only a few large fitness landscapes have been quantified. For example, Pitt et al. measured the fitness landscape of 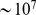 RNA sequences with an in vitro selection protocol [23]. Similarly, Mora et al. studied frequencies of genetic sequences of IgM molecules in zebrafish B cells (which are related to fitnesses), but they imposed a translational symmetry of the sequence [24]. Finally, Hinkley et al. analyzed 70,000 HIV sequences and their *in vitro* fitnesses, built a fitness landscape defined on different amino acids of certain HIV genes, and then investigated large scale properties of the ensuing landscape [25], [26]. However, even in these high throughput studies, the data did not contain all possible pairs of mutations, potentially biasing the results, especially far from the wild type sequences (see *Discussion*).

In this article, we reconstruct a large, yet detailed bacterial genotype to phenotype map, including quantifying the epistatic interactions in the ensuing fitness landscape. We seek a landscape based on long *nucleotide* sequences, which additionally allows quantifying phenotypes of transcriptional regulation in addition to those of enzymatic activity. This permits fitnesses to be defined over both coding and non-coding DNA. To map the landscape far from the wild type genotype, we would like sampling of the sequence data that is unbiased by selection.

Recent experiments by Kinney et al. [27] have collected a dataset that comes close to satisfying these criteria. The data consists of mutagenized transcriptional regulatory sequences from the *E. coli* (MG1655 and TK310 strains) *lac* promoter. In total, there were 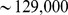 *lac* promoter sequences mutagenized in a 75 nucleotide region containing the cAMP receptor protein (CRP) and RNA polymerase (RNAP) binding sites (−75∶−1), with 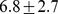 mutations per sequence (mean 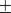 standard deviation) (see Ref. [27] for additional data set details). The transcriptional activity induced by the mutagenized promoters was measured through fluorescence of the transcribed gene products and FACS sorted according to the transcriptional activity into up to nine logarithmically spaced categories. All categories were then independently sequenced, so that the quantitative (on the scale of 1 to 9) phenotypic effect of each sequence is known to within a certain accuracy. Further, there were an additional 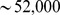 sequence-expression pairs for the same operon analyzes in different environmental conditions. Thus the data can be used to reconstruct the genotype-to-phenotype map. However, the promotor activity is directly related to lactose metabolism and thus is correlated with growth rate or fitness under conditions where lactose is the preferred energy source. Therefore, the fluorescence may also be viewed as a proxy for fitness of this sequence.

In summary, the Kinney et al. [27] dataset provides simultaneous measurements of sequences and their phenotype. Crucially, the data set is dense, so that every pair of mutations has occurred at least 20 times, each time in a different genetic backgrounds of about 5 other random mutations. We use these sequence and transcriptional activity data to infer the detailed genetic landscape for the 75 nucleotide DNA sequence, quantifying pairwise epistatic interactions among all of the nucleotides to the accuracy afforded by the data. This is done by constructing a linear-nonlinear regression model that connects sequences to their phenotypes. Since the number of possible epistatic interactions is comparable with the number of sampled sequences, we control the complexity of the models by 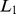 regularization, and hence prevent overfitting. This also imposes sparsity on the epistatic interactions, which we expect from the limited number of binding sites. We then analyze the statistics of epistatic effects in the inferred landscape. Finally, analysis of the landscapes obtained under different environmental conditions provides evidence that the wild-type sequence of the *E. coli lac* promoter is close to optimal in the ecological niche that the bacterium occupies.

## Results

### Inferring the non-epistatic genotype to phenotype map

The simplest model of a genotype to phenotype map is one where each locus contributes a fixed amount to the phenotype, regardless of the state of other loci. Thus we used the sequence and the fluorescence measurements (see *Methods*) to fit an additive map using linear regression of the fluorescence values 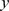 (integers 1 to 9) on the genetic code which are treated as 75 categorical variables with four levels: A,T,G,C. The dummy variables encode the presence of mutations relative to the wild type (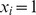 when a mutation is present, and 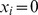 otherwise). Since there are four nucleic acids, each locus has three binary numbers for each of the possible mutations from the wild-type, and the sequence length is effectively tripled. In other words, for each locus, 000 represents the wild-type, and 001, 010, 100 represent the three mutations (see Table 1 in *Methods*). The statistical model is
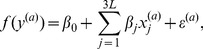
(1) where 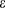 is the statistical noise, and the superscript 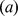 stands for a single bacterium, for which the sequence, 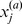, and the fluorescence, 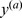, are known. In subsequent equations, the superscript is suppressed for brevity. Part of the genotype-phenotype map may be non-linear due to the mapping from fluorescence to bin number and due to some remaining background fluorescence. Thus we replace 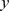 with a non-linear monotonic function 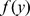 chosen to optimize the explanatory power of the nonepistatic statistical model, and likely bias downwards inferred effects of epistatic contributions (see *Methods*). The coefficients, 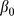 and 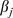, are found by ordinary least squares regression, e. g., coefficients that minimize 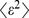 in Eq. (1). Since the wild-type is a sequence of all zeros, 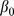 is the predicted phenotype of the wild type. The coefficients can be found in File S1.

**Table 1:** Mutation encoding scheme (dummy variables).

	A	T	C	G
A		100	010	001
T	001		100	010
C	010	001		100
G	100	010	001	

For a wildtype nucleic acid (vertical) a mutation to another nucleic acid (horizontal) is encoded by the corresponding sequence.

The coefficient 
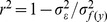
 measures the goodness of fit, or how much of the variance in the data, 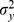, is explained by the model. The linear model yields 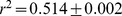.

Some variation in the data is experimental noise, such as background fluorescence and cell-to-cell variability, and sets an upper bound on the possible 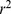. In *Methods*, we estimate this *intrinsic* noise to be 10–24%, and therefore about 76–90% of the total variability of the data can be explained by *any* statistical model, even an arbitrarily complex model. Therefore the linear model accounts for 57–67% of the explainable variance. We emphasize that this statement is not about mechanistic underpinnings of the genotype-to-phenotype relation, but about statistics of the data only. As in any multivariate model, it is possible for the statistical linear effects to emerge from superposition of many mechanistic epistatic interactions.

**Figure 1:**
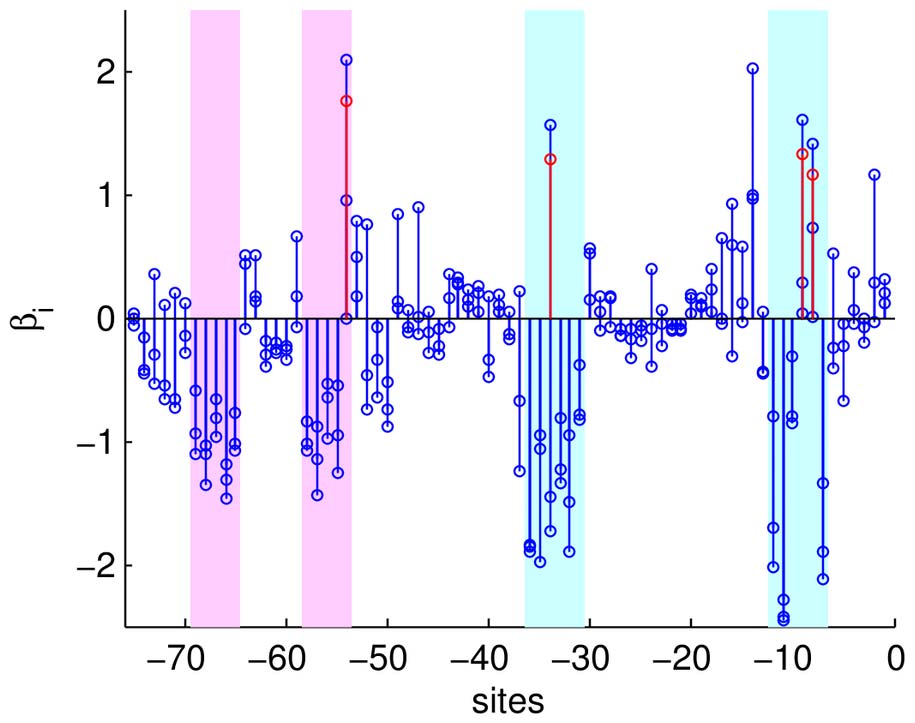
Stem plot of the linear coefficients. Three circles on each stem represent the changes in phenotype for each of the three possible mutations per site. CRP and RNAP are known to each bind at two sites (magenta and cyan areas). Red circles correspond to the mutations needed to get the consensus sequences.

Examination of the coefficients 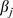 with the largest magnitude reveals the consensus locations of the CRP and RNAP binding sites (Fig. 1), which validates the modeling approach. Interestingly, the wild type does not contain the “consensus” binding sequences: 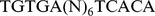 for CRP [28] and 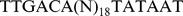 for RNAP [29], but the wild type is only four mutations away. Four of the large positive coefficients in Fig. 1 (positions −54, −34, −9, −8, red circles) correspond to the mutations needed to get the consensus sequences.

These inferred coefficients may be compared to the energy matrices derived from the same data with information theoretic techniques by Kinney et al. [27]. There the energy matrices were inferred separately for CRP and RNAP, and also over many different experiments, while our regression coefficients were inferred from the whole sequence data. Correlation between our 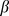
's and the energy matrices ranged from 89%–91% for CRP binding sites. This is comparable to the 95% correlation among energy matrices estimated from different subsets of the data in [27]. Such an agreement between a manifestly simple linear-nonlinear model and the results of a computationally complex optimization of information-theoretic quantities is truly surprising and encouraging.

Since correlations among various energy matrices for the RNAP binding are somewhat lower (92%) [27], we expect the agreement between the regression and the information-theoretic methods to be worse for this case. Indeed, the correlations between 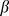
's and energy matrices range between 46% and 54%. We expect that this reduction can be attributed partially to the fact that the energy matrices were inferred by Kinney et al. for CRP and RNAP separately or jointly in a *thermodynamic* model, which assumed a direct relation between RNAP binding and the transcription rate. It has been discussed and measured repeatedly [30], [31] that transcription rate is strongly affected by kinetics of transcriptional initiation, which is not modeled for by the thermodynamic probability of finding RNAP bound to the regulatory sequence. Unlike the energy matrices, our statistical model inferred from the entire sequence can account for these kinetic effects, and may be more accurate in this context. Since such effects are absent for transcription factor binding, they can potentially explain the differences in agreements between the models observed for CRP and RNAP binding sites. Such kinetic effects may also explain the difference between the wild type and the consensus (that is, the strongest) binding sequences mentioned above. Additional biophysical experiments are needed to carefully explore these issues.

### Inferring epistatic contributions to fitness

The simplest model with epistatic interactions between all pairs of nucleotides is a quadratic or bilinear model, written as:
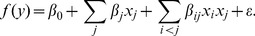
(2)


The last sum is over all nucleotide pairs. Here nonzero 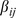 would indicate the presence of pairwise epistasis. For example, 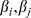, and 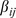 all of the same sign is comonly referred as synergistic epistasis, where contribution of the pair of mutations is stronger than of each mutation alone. Other possible types of epistasis are described below.

Note that, in Eq. (2), we keep 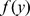 the same as in the previous section, which maximizes the explanatory power of the non-epistatic terms and minimizes that for the epistatic terms. The number of epistatic terms in this statistical model (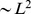) should be contrasted with typical biophysical models of protein-DNA interactions, which include only a single free energy term describing interactions between the CRP and RNAP proteins [27], [32].

The total number of coefficients 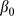, 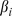, and 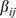 in the quadratic epistasis model, Eq. (2), is 25,201 (accounting for the fact that, in a single genome, only one mutation per site is allowed). Overfitting is a concern since the number of observations, 129,000, is not much larger than the number of coefficients. To infer a model that does not overfit, we applied a standard regularization procedure, which penalizes overly complex models and imposes sparsity on the number of nonzero interaction terms (see *Methods*). Since available genotypes were not uniformly distributed, but rather biased towards the wild type, we supplemented traditional cross-validation approaches with additional checks to ensure that the regularization selects the model with the highest explanatory power, but no overfitting. The chosen model and its coefficients are discussed in the following. Coefficients of the chosen model, the full model, and the model deemed best by cross-validation can be found in File S1. As we show in *Methods*, the general structure of the inferred epistatic coefficients 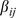 is only weakly dependent on the specifics of the model choice.

**Figure 2:**
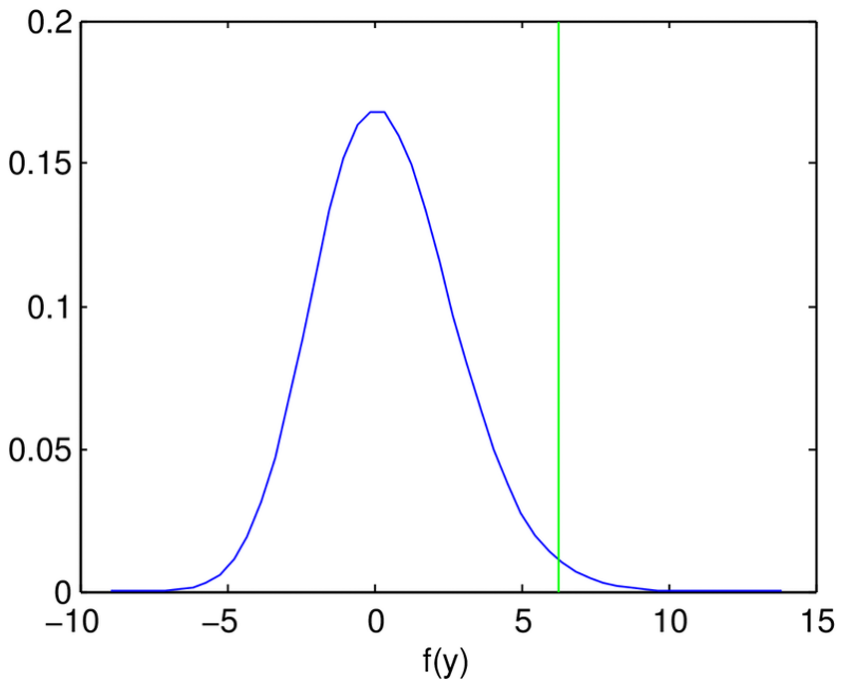
Histogram of phenotype 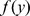 values of 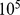 uniformly random sequences for the inferred epistatic model. Random sequences have very low inferred phenotype values because of the specificity of binding sites. The peak of the distribution indicates what phenotype values evolve under neutral conditions. The the wild-type value, 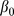 (green line), is much higher than the neutral value indicating selective pressure.

The distribution of inferred phenotype values for randomly generated sequences (Fig. 2) shows that the random sequences are typically not very functional (presumably because the binding sites loose specificity). The peak near 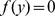 represents the most common sequence that would be observed under neutral evolution, and the relatively high value for the wild-type (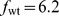) compared to the random sequences indicates that it is under strong selection. Notice that we can assert this without any comparative genomics or population genetics data, which would typically be required.

The fraction of variance explained by the pairwise epistatic model is 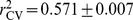 (although it is sensitive to the regularization parameter, see *Methods*). Comparing to the non-epistatic model with 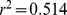, and taking into account the intrinsic experimental noise of 10–24%, we see that about 7% of the explainable variance is due to the pairwise epistasis. However, it is possible that more data would increase the amount of predictive power of the epistatic contributions. Furthermore, combinations of multiple epistatic interactions may have a net nonepistatic contribution to the phenotype (but not the other way around). Thus this 7% figure is, in many respects, a negatively biased estimate of importance of epistasis.

**Figure 3:**
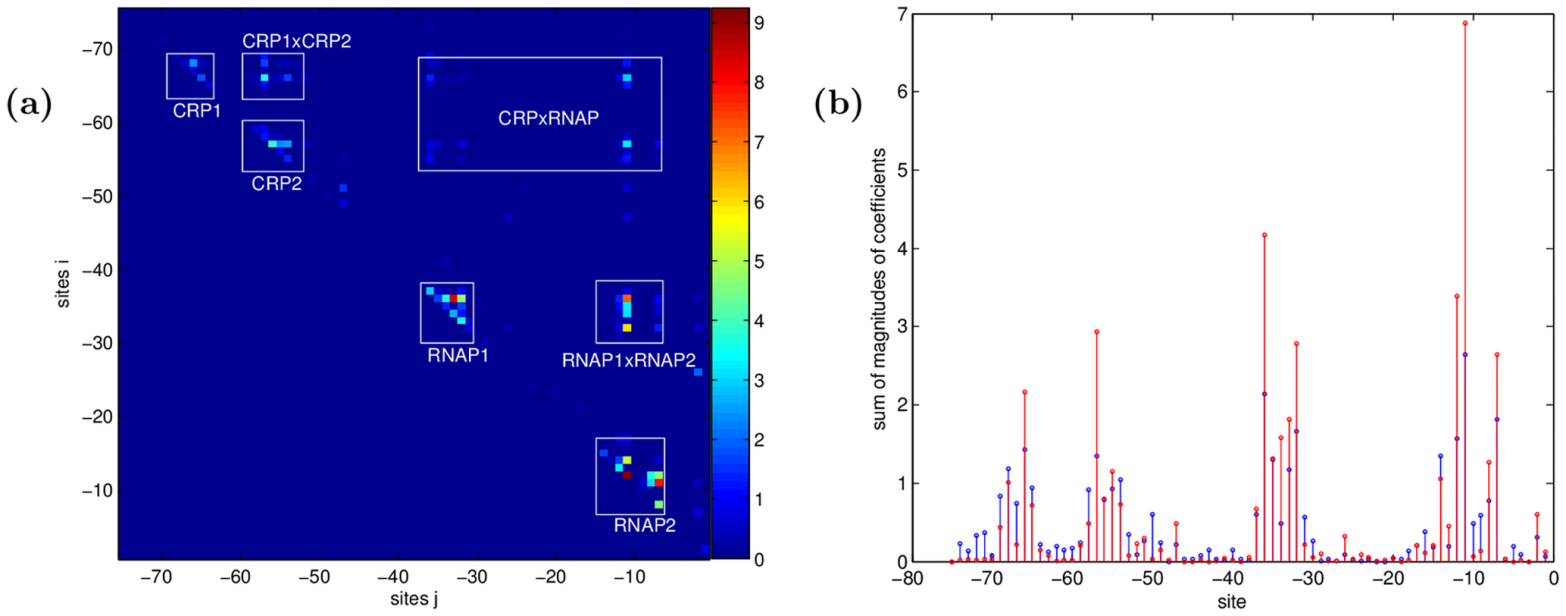
a) Matrix of the sum of the absolute values of the pair interaction coefficients for each pair of sites 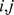 (3 mutations per site equals 9 interactions) for the chosen statistical model. The clusters near the diagonal are interactions within the RNAP and CRP binding sites, and the off-diagonal clusters are interactions between the binding sites. b) Red: Site-specific sum of absolute values of additive coefficients, divided by 3 (the number of possible mutations). Black: site-specific sum of absolute values of epistatic coefficients, divided by 9 (the number of possible mutation pairs). Epistatic and additive effects are strongly correlated, with the correlation coefficient 0.90.

The non-epistatic coefficients are about 70% non-zero, but the interaction terms are very sparse, about 3% non-zero. The phenotype is affected by mutations in some positions more than others. Coefficients with the largest magnitudes belong to positions within the CRP and RNAP binding sites (see Fig. 3). Thus this kind of data allows for identification of binding sites without a biophysical model of protein-DNA interactions, as is done traditionally [33], [34]. More importantly, as Fig. 3 shows, the model can infer functional interactions between amino acid or nucleic acid binding over a much longer range than can be computed from biophysical and structural biology approaches [35]. The consistency of our results with known binding sites validates our inferences. Alternative methods that instead limit the number of inferred coefficients by constraining the range of interactions, or by allowing interactions only between consensus sites, would either miss the long-range effects, or the small (but statistically significant) interactions away from the binding sites seen in Fig. 3.

The interaction coefficients are observed to be clustered around the subunits of the system CRP, RNAP, and their constituent binding sites. The inter- and intra- binding site interactions are easy to separate in Fig. 3, allowing a comparison of the magnitude of the interactions between the subunits, summarized in Tbl. 2. Interestingly, CRP and RNAP interact on the same order of magnitude as their constituent binding sites interact among and within themselves.

Epistatic interactions may be classified into several categories (see Table 2): synergistic epistasis (the effect of two same-sign mutations is larger than the sum of the effects of each one separately), antagonistic epistasis (the effect of two same-sign mutations is smaller than the sum of their individual effects), and other epistatic effects (the individual effects of two mutations have opposite signs, while epistasis is present). We find that most of the interactions in the *E. coli lac* promoter are antagonistic (388/629 = 62%). This is likely because mutations change protein-DNA binding affinity nearly additively, which leads to “diminishing returns” from contributions of individual mutations to transcriptional activity, similar to [4], [6]. Indeed, if the transcription rate is given by a sigmoidal function of the binding free energy 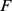, such as 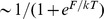 or similar [27], then improvements in 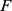 are incrementally less important when it is already large and negative. Thus the effect of matching an appropriate nucleotide to the corresponding amino acid decreases when other bases are already matched. Epistasis produced by this mechanism should be antagonistic, but mild [4], [6]. Indeed, we found only one case of a severe type of antagonistic epistasis (reciprocal sign epistasis), where the individual effects are both harmful, but the total effect is beneficial. It is known that reciprocal sign epistasis is a necessary (but insufficient) condition for a multi-peaked landscape [36], and hence we expect this landscape to be fairly smooth (at most two maxima).

**Table 2:** The interaction coefficients for 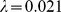 are clustered around the subunits of the system: CRP, RNAP, and their constituent binding sites (defined by white rectangles in figure 3a).

	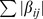	non-zero	antagonistic	synergistic	sign
all	194	629	388	56	185
CRP1	8.2	43	36	1	6
CRP2	16.1	58	26	5	27
CRP1×CRP2	14.5	77	54	4	19
RNAP1	36.8	75	58	5	12
RNAP2	49.7	88	31	1	56
RNAP1×RNAP2	29.8	82	64	9	9
CRP×RNAP	25.4	128	115	1	12

The total amount of interaction (sum of the magnitude of coefficients) is shown in the first column. The interactions are categorized into three exclusive types of epistasis: synergistic, 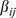, 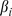, and 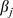 share the same sign (and are non-zero), antagonistic, 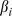 and 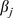 share the same sign, but 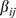 has opposite sign, and sign epistasis, 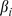, and 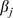 are of opposite sign and 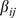 is non zero.

While the relationship between phenotype (transcription) and fitness is not precisely known in this experiment, they are likely to be correlated. Therefore the roughness in the genotype-phenotype map is likely to be important for the whole fitness landscape. Identifying fitness with 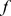, we characterized this roughness by directly exploring the accessibility of the local optima of the inferred map. We used an adaptive walk similar to the evolution of a large population in the weak mutation regime, which can move only towards higher values and cannot escape local maxima. Starting from the wild-type sequence, the algorithm only chooses mutations that increase the phenotype (or fitness), with probability proportional to the log fitness difference. Out of 1000 random walks, the population ends up in only two very similar sequences which differ by 2 mutations, and they are 40 and 39 mutations away from the wild type (compare to the average of 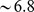 mutations per sequence). Since the sequences are so far away from the training data, their predicted phenotype value are not accurate predictions of the real local maxima.

### Second and higher order epistasis for a subsequence

We have insufficient data to study third and higher order epistasis on the entire 75 bp sequence. However, since most of the linear and the 2nd order epistatic effects in our analysis are concentrated at the consensus binding sites (cf. Fig. 3), we have performed 3rd order epistatic analysis on 22 base pairs subsequences of the data, limited to the four known binding sites in the sequence. That is, in addition to the linear and the bi-linear model, we also fitted:
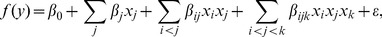
(3) where the same procedure was used to find the non-linear function, 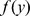 (see *Methods*). Note that the 22 base pairs were selected based upon consensus binding site locations, not upon our analysis in the preceding sections. Thus one does not expect overfitting that would ensue if the same data were used to identify the binding sites first, and then to refine their epistatic model.

For this subset of nucleotides, the model with only additive effects, Eq. (1), had an 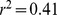. The 2nd order epistatic model, Eq. (2) had 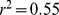. Here the number of interaction coefficients was much smaller (2,212), resulting in no signs of overfitting even without regularization. Thus the importance of quadratic epistasis, which explains 14–20% of the explainable variance for the subsequence, is no longer data limited. Like for the full sequence, we investigated the roughness of the landscape created by the binding sites subsequence. We found the landcape to be smooth, with only one global maximum, exactly matching the consensus (but not the wild type) regulatory sequence.

The 3rd order epistatic model, Eq. (3), had 47,972 coefficients, which needed to be regularized in the same way as the quadratic model (*Methods*). This yielded 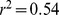 at maximum cross validated 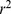. Thus the higher order interactions do not improve the fit, and there is *no evidence* for these 3rd order epistatic interactions in the data, although it is possible that larger data sets would reveal them. Similarly, further restricting the subset of base pairs used in the analysis did not discover statistically significant 3rd order effects. In other words, quite surprisingly, for these data, combinatorial *effects of triple mutations can be fully modeled by effects produced by constitutive pairs of the triples*.

### Landscape in two environments

In addition to the data from the three experiments analyzed above, Kinney et al. [27] performed experiments with a different strain of bacteria (TK310) that is unable to control its intracellular cAMP levels. Because CRP is activated by cAMP, varying extracellular cAMP levels controls the active intracellular concentration of CRP. *E. coli* prefers to metabolize glucose over lactose, so cAMP is inhibited by the presence of glucose, and *lac* expression is suppressed when glucose is present. We inferred genotype-phenotype maps using the non-epistatic model as in the Section 2.1 for two conditions, no cAMP and 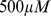 cAMP, representing an environment with glucose and no glucose. The datasets are smaller (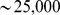 sequences), and distinguish only 5 levels of fluorescence, but they are otherwise very similar, so the same linear-nonlinear 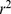 optimization was used. The results shown below were found with the non-epistatic model. However, here the pair interactions account for a smaller fraction of the variance, and the epistatic model produces very similar fitted values.

**Figure 4:**
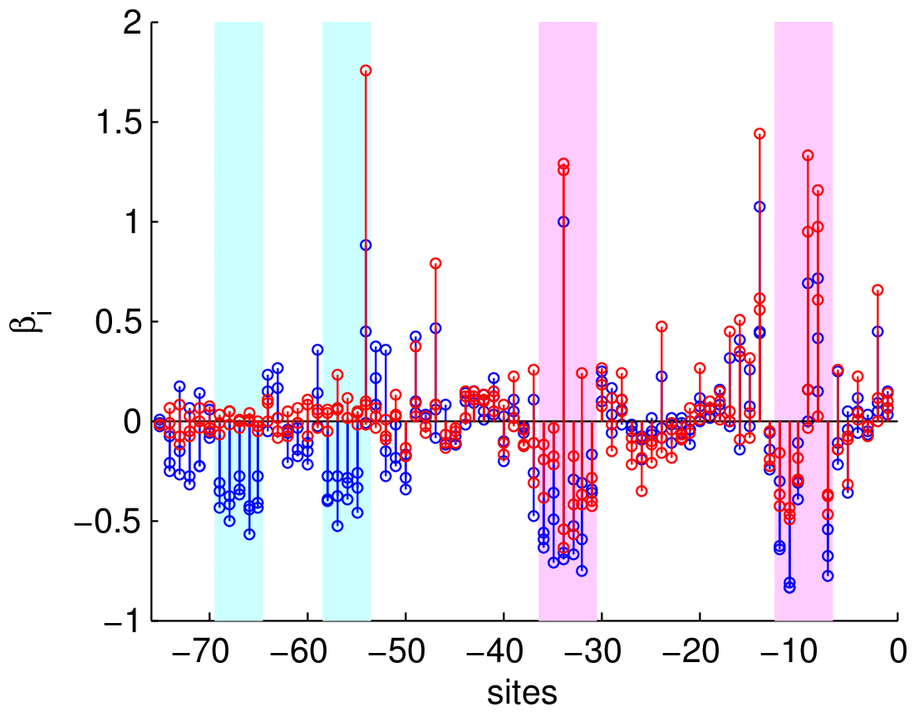
(blue) coefficients 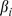 for the non-epistatic model with no-glucose (normal levels of cAMP) (red) with glucose (no cAMP). CRP is activated by cAMP and does not bind without it.

**Figure 5:**
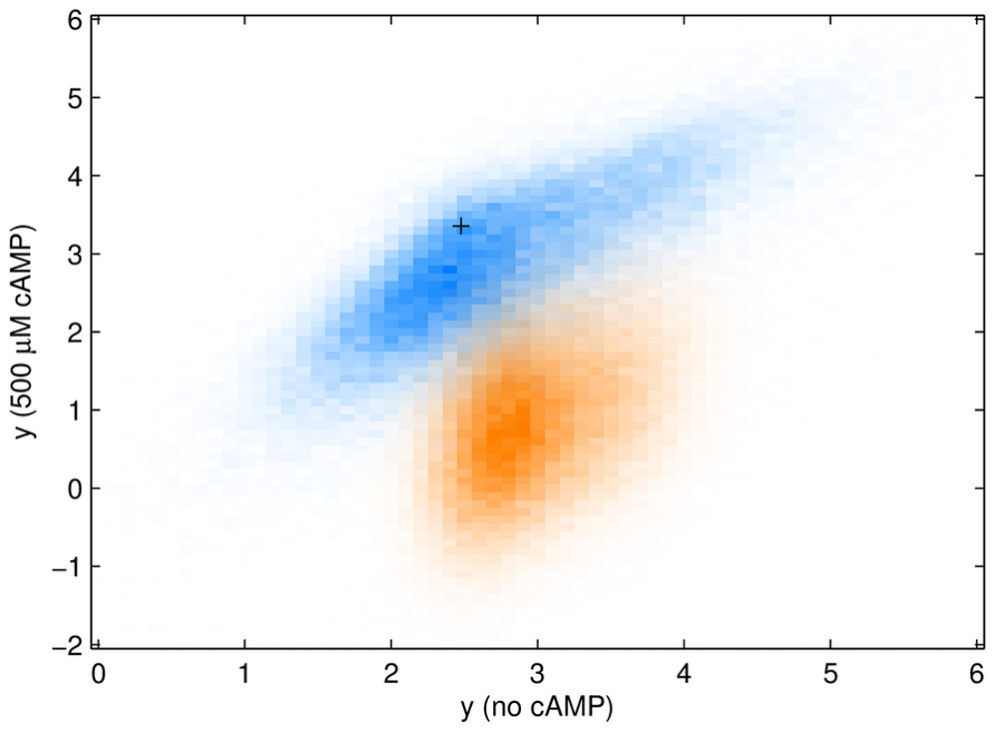
2D histogram of expression for the two environments, no cAMP (glucose), and cAMP (no glucose) for 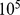 random sequences (orange), and sequences from the experiment (blue), which are closer to the wild type (plus sign). The wild-type is nearly on the optimal front in that very few sequences have both higher expression with cAMP and lower expression without cAMP (above and to the left of the plus sign). The phenotype values range from 1 to 5 in these experiments. The dis-similarity of measured expression and expressions predicted for random sequences along the vertical, but not the horizontal axis, likely signals presence of poorly understood biophysical mechanisms differentially employed in the two considered environments.

As expected, when CRP is not active there is little binding at the CRP sites, and the associated coefficients are almost all small (Fig. 4). Because of the lack of CRP binding, expression for the wild type sequence, and sequences close to the wild-type, is lower when there is glucose (Fig. 5). However, there are some changes to the RNAP binding site coefficients. Random sequences are not functional in the no-glucose environment, but they have some small functionality, comparable to the wild-type, in the glucose environment (Fig. 5), suggesting that there is less specificity in the RNAP binding. Note also that some of the coefficients, especially for the no cAMP case, are large just outside the traditional RNAP binding domain. Unexpectedly, for no cAMP, the transcription rate is comparable to the cAMP present case, when CRP helps polymerase recruitment. This suggests some additional biophysical binding mechanisms, currently unexplored. As discussed above, these mechanisms are quite possibly kinetic in nature.

In the no cAMP (glucose) environment, *lac* expression should decrease the growth rate because the cell is metabolizing glucose instead of lactose, and *lac* expression costs resources [37], [38]. Therefore we expect sequences under selection, such as the wild type, to have relatively high expression with cAMP, and low expression without cAMP, compared to sequences not under selection (random sequences). Figure 5 shows that there exist very few sequences which are better than the wild type in both environments, i.e. simultaneously higher expression with cAMP, and lower expression without cAMP. The non-elliptical shape of the fitted values for the experimental sequences suggests again that the wild type is under a strong selection towards the top left corner of the plot. Finally, we point out that, even when lactose is being metabolized, too high expression of *lac* genes is costly, possibly because cellular resources are pulled to *lac* transcription and translation and away from production of essential proteins [37]. This may make sequences in the top right corner of Fig. 5 less fit than our monotonically increasing 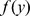 model assumes, making the wild type even closer to the global optimality.

## Discussion

We constructed a genotype-to-phenotype mapping, including effects of all pairwise and some higher order epistatic interactions. This was done by analyzing functional properties of over 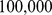 randomly mutated sequences in the vicinity of the wild type *E. coli lac* operon, queried under different experimental conditions. The control of dimensionality for the epistatic models, along with the large size of the dataset, allows for a much more detailed analysis of epistasis in this bacterial genetic regulatory region.

Our approach is generally similar to those in Refs. [25], [26]. However, there are substantial differences beyond a different model organism used. Our alleles are nucleotides in a regulatory region of a bacteria, instead of amino acid variants. Our landscape is more complete, in that interaction among all pairs of nucleotides in the sequence are estimated from the data that includes each such pair at least 20 times in different genetic backgrounds. In particular, we have relaxed the condition [24] that the interaction terms 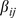 can depend only on the distance between the loci, rather than on the specific positions of the loci. Mora et al. [24] used maximum entropy approaches to infer a fitness landscape, while, along with Hinkley et al. [25], we have focused on linear regression (though with different regularization constraints and different nonlinear mapping between the fitness and the observed phenotype). The epistatic model, Eq. (2), is the same in the regression and the maximum entropy approach. However, the philosophical basis behind the approaches is different, and so are the criteria used to specify the coefficients 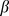. Maximum entropy methods choose them to constrain observable correlation functions, while regression attempts to approximate the entire fitness function. It remains to be seen which of the two frameworks provides a better model for genomic data.

Possibly the largest difference from the previous approaches that considered epistatic interactions for many mutations is that we found a genotype-phenotype map, rather than the true fitness landscape. While we expect the phenotype and the fitness to be strongly correlated when lactose is being metabolized (and anti-correlated otherwise), the relation between the fitness and either the observed fluorescence or its nonlinearly reparameterized form, 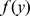, is likely nontrivial. Ideally, a second experiment would measure the phenotype-to-fitness map to complete the reconstruction of the fitness landscape. In fact, Dekel and Alon[37] have completed this second step for the *lac* regulatory sequence. However, we cannot use their findings since their *E. coli* strains and growth environments were slightly different from those of Kinney et al. [27].

Binding energy-fitness maps have been inferred from genome wide studies of transcription factor binding sites using genomic statistics and population genetics models [39]–[42]. In those studies, the genotype-phenotype maps were largely assumed to be non-epistatic, in contrast to our work. It would be interesting to combine the methods to make a more complete account of epistasis from genotype to fitness.

Our observations have revealed a few cautionary notes regarding using genome frequency in a population to reconstruct fitness landscapes [24], [25]. In such experiments, all sequence data (including whatever part of it that is left for cross-validation) are localized near the wild type, near-optimal sequences due to selection. Carefully inferred models (whether regression or maximum entropy based) perform well for the observed data, but will generalize badly for sequences far away from the wild type. Our approach samples the genotype space more evenly without selection, and therefore is better suited for making inferences about the global landscape properties, such as its ruggedness. Nonetheless, even in our data, with each sequence 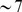 mutations away from the wild type, extrapolation to much larger genotypic differences produces absurd results, even if cross-validation fails to notice problems, (see *Methods: Regularization and model selection*.

In our inferred landscape, epistasis accounted for about 7% (about 15% for the binding sites subsequence) of the explainable variance. Most of the epistasis was antagonistic, but the landscape was essentially single peaked. This is similar to properties of epistasis in metabolism [4], [6], and the explanation for both likely involves diminishing returns from successive individual mutations. It is useful to contrast these findings with the work on HIV [26] or protein fitness landscapes [7], which have observed more substantial epistasis and many more local maxima. While it is possible that more epistatic effects would be observed for our system if more data were available, more intriguing is the following observation. During model selection (see *Methods*), it was noticed that, due to most of the sequences being 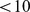 mutations from the wildtype, it was possible to make large prediction errors for sequences with more mutations. In other words, there was a large extrapolation error for sequences outside of the training data, and this led to choosing a more constrained model for final analysis. A less constrained model (which maximizes 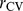, cf. *Methods* *: Regularization and Model Selection*) is much more epistatic, with adaptive walks indicating many local maxima. The severity of the problem correlates with the nonuniformity of the genotype sampling, making the data from populations under strong selection especially suspect. To allow studying global properties of landscapes, an ideal experiment would sample the sequence space much more uniformly to avoid extrapolation.

In addition to the weak epistasis, we also found that the wild-type *E. coli lac* regulatory region is optimal for the two environments measured. That is, it is on the front of possible sequences which maximize expression when it is beneficial, and minimize expression when it is harmful. If under the growth conditions the fitness is a non-monotonic function of the transcriptional activity and decreases at high expression [37], the wild type operon may be not only nearly multi-objective optimal, but nearly globally optimal. To investigate this, experiments are needed that would study fitnesses of many sequences under selection in fluctuating environments.

The ability of our method to identify protein binding sites and epistatic interactions among them raises an important point. These epistatic interactions, inferred by either of the methods we have mentioned in this work, especially interactions over long ranges, may not correspond to true biophysical interactions between amino acids and nucleotides. They are likely *effective* interactions resulting from collective effects of many other epistatic terms, including higher order terms, or a small number of interactions, such as binding between CRP and RNAP. While there is an admirable similarity between our linear regression coefficients and energies of protein-DNA interactions, our approach may not be as informative where there is enough information to build a detailed biophysical model, but there are few places in the genome where this is the case. On the other hand, our approach can detect long distance epistasis, or non-thermodynamic effects on transcription where a priori it is unclear that these effects and interactions exist. When working on the genome scale, effective models that can make accurate *predictions* of phenotype or fitness for previously unobserved sequences may be useful regardless of their lack of microscopic accuracy. They may be closer to the right level of description of the problem [43], by striking a balance between microscopic biophysically relevant detail, and power to describe the richness of phenomena emerging on the genomic scale. As an example of this utility, here we found that, for the 22 bp long subsequence of the regulatory region that includes the binding sites, there was no evidence for 3rd order epistatic effects. The fact that pairwise *effective* interaction models, with only a few higher order contributions, provide excellent fits to multivariate data has been observed by now in the context of neurophysiological recordings [44]–[48], microarray-measured gene expressions [49]–[51], and sequencing data [24], to which our analysis has just added another example. These frequent successes of pairwise models in diverse domains are certainly surprising and, as of now, unexplained. They raise many interesting questions about general theories of multivariate biological data, which are still waiting for their answers.

## Methods

### Preparation of the dataset

To make inferences on the largest dataset possible, we combined the data from three experiments done by Kinney et al. [27] (fullwt, crpwt, rnapwt, 129,000 sequences total), which differ only by the regions in which mutations were allowed to take place. Fullwt was mutagenized over the whole sequence (−75∶−1), while crpwt and rnapwt were mutagenized only over the CRP binding area and RNAP binding area. In addition, some sequences were rejected for data quality reasons: identical sequences in the same bin were likely to be not independent measurements (see Supplemental Materials in Ref. [27]), and sequences with an exceptional number of mutations (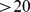) were probably errors.

### Linear-nonlinear model

Part of the genotype-phenotype map may be non-linear due to the mapping from fluorescence to bin number and some remaining background fluorescence. To identify pairwise interactions in the background of an arbitrary mean nonlinear genotype-phenotype map, we introduce a generalized linear-nonlinear model:
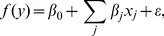
(4) where 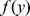 is a monotonically increasing, nonlinear function of 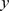. The function is found by maximizing the fit (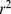), which corresponds to minimizing
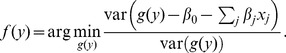
(5)


**Figure 6:**
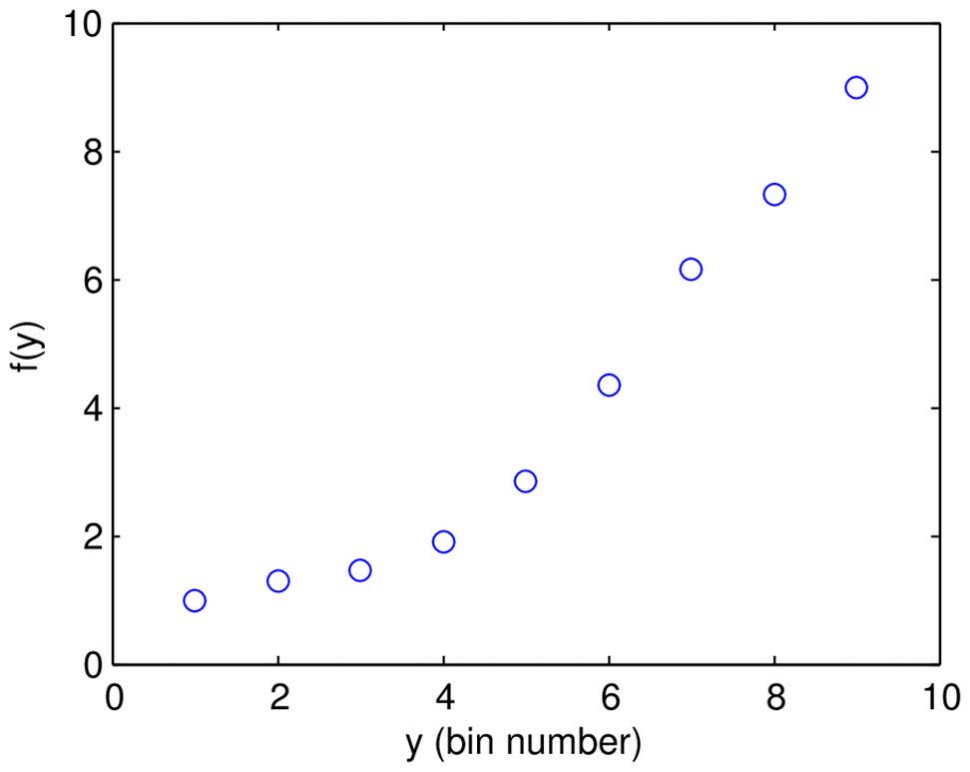
Generalizing the fitted function by replacing the output values 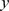 with a non-linear function 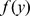 improves the least squares fit. Constrained non-linear optimization found the optimal 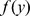 for the linear model with 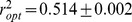. The non-linearity is due to the first few bins being dominated by background fluorescence and not gene expression.

We add the constraints that 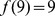, and 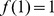 to keep 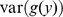 finite. The function 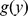 is defined over only 9 values of 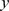, and a constrained non-linear optimization procedure (fmincon from MATLAB) finds an optimal 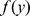 quickly (Fig. 6). Note that this method resembles a type of generalized linear model called ordinal probit regression[52], and is also similar to the inference of non-linear filters in computational neuroscience using information-theoretic tools [53].

The summary statistics change when replacing 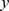 with 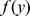. The variance of the bin numbers increases from 6.5 to 7.6, and the 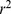 increases from 0.476 for the linear model for 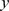, to 0.514 for the linear model for 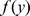. The experimental noise estimates (see below) are also slightly different.

Assuming a monotonic relationship between genotype and phenotype, 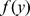 is the function that maximizes the phenotype prediction from the non-epistatic (linear in 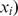 contributions. This reduces the amount of variability left to be predicted by *any* epistatic model, whether of genotype-phenotype map, or genotype-fitness map (provided that the fitness is monotonically related to the phenotype). This also prevents the epistatic model from fitting any average non-linear effects. Thus our subsequent assessment of importance of the epistasis should be viewed as biased towards underestimation.

### Estimates of intrinsic noise in the data

Experimental data is corrupted by errors in both fluorescence measurements and sequencing. One estimate of this intrinsic noise is obtained by averaging the variance of 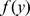 for identical sequences with different recorded fluorescence values. The ratio of this intrinsic variance to the total variance of 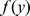 is 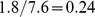. Since this excludes all sequences that fell into just one bin and have an unknown variance 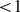, this estimate is an upper bound on the noise variance.

Another estimate can be obtained by using the controls from Ref. [27], which provide fluorescence numbers for many individual wild type bacteria. The fluorescence variance in optimized bin units is 0.74, which is 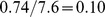 of the data variance. This number underestimates the average noise since wild type bacteria express strongly, so that the fluorescence noise for them is smaller than for most other sequences.

### Regularization and model selection

Statistical model with the number of parameters comparable to the data set size may overfit, that is, model statistical noise in the data. To prevent overfitting, we minimize the mean squared error in Eq. (2) subject to a regularizing constraint
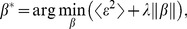
(6) where 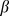 is the concatenated vector of all the regression coefficients, 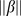 is its norm, and 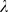 is a free parameter (Lagrange multiplier), unknown *a priori*. Regularization constrains the statistical complexity of the model by minimizing the norm of the coefficients [54]. When the 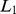 norm is used, 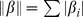, this regression is called the Least Absolute Shrinkage and Selection Operator (LASSO) [55]. LASSO favors sparse solutions, which is a reasonable assumption since most of the 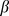
's are interaction terms, and interactions are presumed to be mainly between the relatively small CRP and RNAP binding sequences. Thanks to an efficient implementation of the algorithm [56], we can compute the LASSO solution for 100 different values of 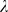, from the maximum value (where the solution is all 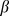
's equal to zero), to four orders of magnitude smaller.

**Figure 7:**
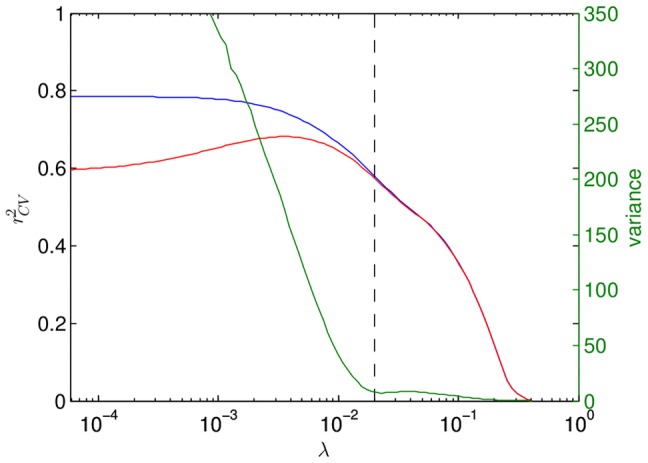
The LASSO solution of the quadratic model was computed for 100 values of 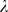. Blue is the 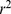 value, and red is the 10-fold cross-validated 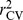. The green curve is the variance of 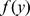 for randomly generated sequences. The variance is too large even for values of 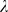 that are larger than the optimal value predicted by the maximum of the 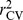 curve. We choose the model with 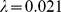 (dashed line) for further analysis. This model has 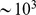 non-zero coefficients, most of which are epistatic.

**Figure 8:**
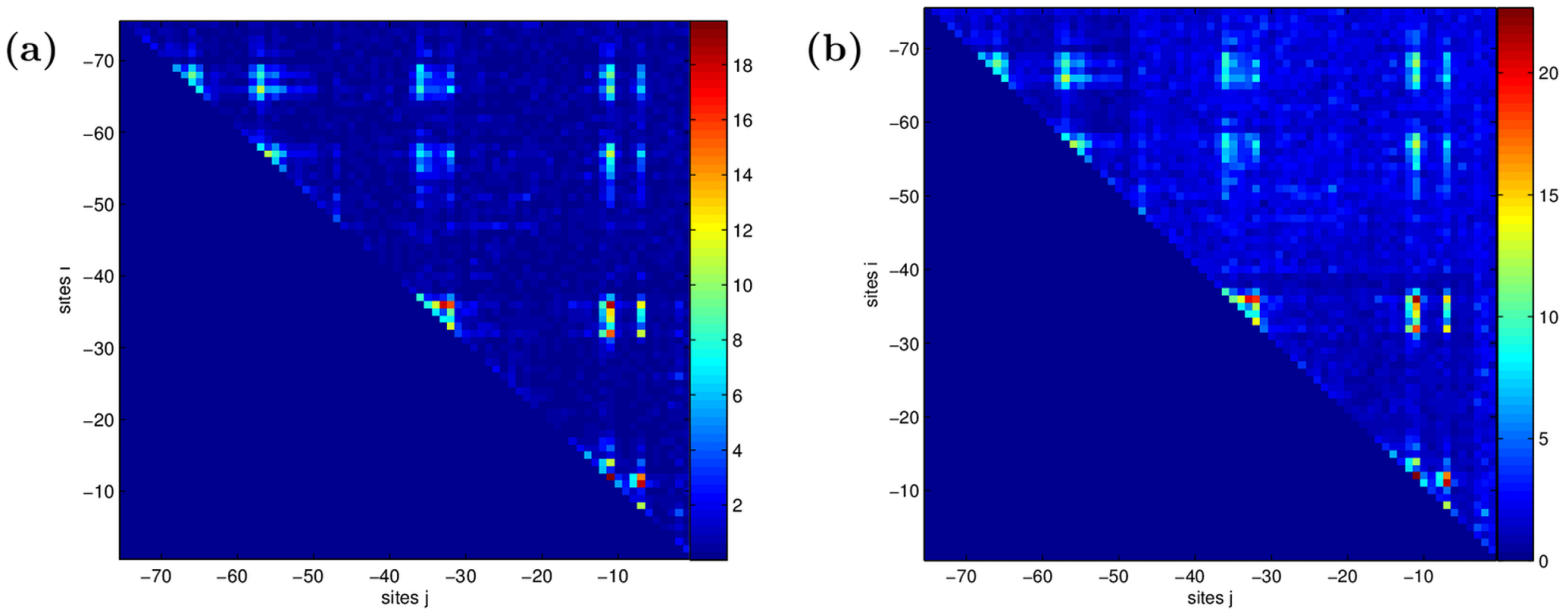
Sensitivity of the epistatic coefficients to the choice of the regularization parameter 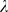. As in Fig. 3, we show the matrices of the sums of the absolute values of the pair interaction coefficients for each pair of sites 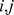. a) Coefficients for the model with maximum 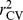 (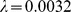). b) Coefficients for the full model: 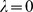. Notice the same general structure of the coefficients for varying 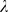, including 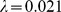 in Fig. 3. This indicates stability under changes of the parameter.

However, choosing the *best* solution (i.e., the right 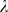) is ambiguous. A common method of model selection is cross-validation. Figure 7 shows that solutions with large 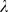 are a poor fit, while small 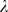 values have less predictive power, as seen through cross-validation. Typically one chooses the best model as the one with the maximum 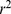 (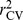) [55]. However, both the training and the cross-validation data are sequences with an average of only 6.8 mutations from the wild-type (9% mutated sites). Thus cross-validation may not ensure predictability for sequences farther away in the genotype space. Indeed, the variance of the fitted values of 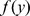 for the experimental data is not sensitive to changes in 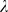 (not shown). Nonetheless, Fig. 7 shows that the variance of 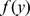 for random sequences blows up for less constrained models (low 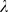), where unrealistically high fitted values of 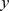 or 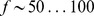 emerge. This indicates overfitting due to uneven sampling of the genotype space and the resulting correlations in the training and the test data. We thus limit 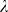 to the range where the variance of the fitted values for random sequences is comparable to that for the experimental data and is insensitive to 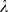. Incidentally, this is also the place where 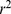 and 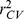 curves split in Figure 7 (dashed line, 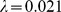, 629 non-zero coefficients). Finally, Fig. 8 shows that the general structure of the solution is only weakly dependent on the exact choice of 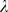.

## Supporting Information

File S1
**Coefficients** 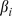 and 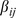 **of the inferred statistical models.**
(XLS)
